# Editorial: The role of toll-like receptors and their related signaling pathways in viral infection and inflammation

**DOI:** 10.3389/fimmu.2024.1363958

**Published:** 2024-01-19

**Authors:** Oliver Planz, Ralf Kircheis

**Affiliations:** ^1^ Institute for Immunology, Eberhard Karls University Tuebingen, Tuebingen, Germany; ^2^ Department of R&D, Syntacoll GmbH, Saal an der Donau, Germany

**Keywords:** TLR - toll-like receptor, viral infection, innate immunity, inflammation, signaling pathway

The Research Topic addresses the role of Toll-like receptors (TLRs) and their associated signaling pathways in viral infections and inflammation. The focus is on how these receptors and pathways contribute to protecting the body against viruses and their involvement in associated inflammatory processes. The present Research Topic encompasses 13 studies shedding light on the role of TLRs in viral infections and inflammation from various perspectives.

The impact of TLRs on viral infections has been elucidated in three studies, effectively illustrating the dual nature of TLR activation in viral infection – both necessary for immune protection and contributing to virus-triggered immunopathology (1–3). Miles et al. demonstrated that TLR7 supports chronic respiratory disease after RSV infection in mice. This study unveils a previously unknown molecular mechanism of lower respiratory tract pathogenesis by RSV, emphasizing the potential of TLR7 modulation to confine RSV pathology to the upper respiratory tract. This restriction prevents its spread to the lower respiratory tract and results in a significant reduction in clinical symptoms.

Theiler’s Virus, known for its ability to infect the central nervous system of mice and trigger an inflammatory reaction, is commonly utilized as a model in research to investigate the mechanisms of virus infections and the course of inflammatory reactions in the brain (4–6). In this study, Kim reviewed the role of TLRs in the mouse model of Theiler’s Virus infection, concluding that the state of TLR activation in the host plays a crucial role in the initial virus replication and persistence.

The use of estrogens in feminizing hormone therapy (FHT) can elevate inflammatory reactions and the risk of cardiovascular mortality in transgender women (TW), who are at an increased risk for both human immunodeficiency virus (HIV) and cardiovascular diseases (CVD) (7–9). Kettelhut et al. demonstrate that *in vitro* data suggest estrogen exposure can enhance the activation of the innate immune system in FHT. The authors discuss the complex interactions of FHT, HIV, and CVD in TW, emphasizing the need for further research to fully understand these interactions and determine optimal FHT regimens or complementary treatments aiming to reduce excessive immune activation.

Lipopolysaccharide (LPS), a TLR4 agonist, is used as a stimulus in many *in vitro* and *in vivo* models (10, 11). O’Neill et al. investigated the impact of dexamethasone on the expression of antimicrobial mediators in LPS-activated primary macrophages, demonstrating the inhibition of both the expression and function of interferon-β. Interferon-β plays a crucial role in the early antiviral immune response.

In a study with healthy volunteers using an imiquimod (IMQ) model, Assil et al. showed that orally administered prednisolone suppresses skin inflammation induced by IMQ, a topical agent that triggers local inflammation through TLR7.

Resiquimod, an imidazoquinoline compound with antiviral and antitumor activity, functions as a TLR7/TLR8 agonist (12, 13). In their work, Keppler et al. point out that clinical use is limited to topical application. Systemic applications of TLR ligands like Resiquimod have failed due to side effects restricting dose and efficacy. Therefore, the authors developed TLR7/8-agonistic imidazoquinolines designed to distribute via endosomes using a macrolide carrier. The substances were designed to distribute to cellular compartments where the target receptor and a specific combination of signaling molecules relevant for IFNα release are present.

The immune reaction to biomaterial implants, known as the foreign body reaction, poses a significant challenge in biomedical engineering, as it can lead to chronic inflammatory reactions to the implanted material (14). McKiel et al. demonstrate that damage-associated molecular patterns (DAMPs) and other intracellular proteins easily adsorb to biomaterial surfaces in competition with plasma proteins. Adsorbed DAMPs in adherent macrophages trigger an inflammatory reaction mediated by the MyD88-dependent TLR2 signaling pathway.

The TLR-2 signaling pathway is also involved in the inflammatory reaction triggered by sperm in the uterus during fertilization, but the precise molecular mechanism remains unknown. According to ligand specificity, TLR2 forms a heterodimerization with TLR1 or TLR6, respectively, as the initial step to mediate intracellular signaling and induce a specific type of immune response. Mansouri et al. aimed to identify the active TLR2 heterodimer (TLR2/1 or TLR2/6) involved in the immunological interaction between sperm and the uterus in cattle. Overall, the results show that sperm utilize the heterodimerization of TLR2/1, but not TLR2/6, to trigger a weak physiological inflammatory reaction in the bovine uterus. This could be the mechanism to remove excess dead sperm remaining in the uterus without tissue damage and create an ideal uterine environment for the uptake and implantation of the early embryo.

In the present Research Topic, there are three publications with a direct clinical relevance. In a case report by Hirsiger et al., the occurrence of agranulocytosis within a few days after the first dose of an mRNA-1273 vaccine against COVID-19 in a previously healthy older adult is described. The patient was diagnosed with large granular lymphocytic T-cell leukemia (T-LGL) of STAT3 wild type. The mRNA-1273 vaccine activated TLR-3, leading to TLR-mediated IL-6/STAT3 pathway activation. The authors changed the vaccination strategy and used a vector vaccine, and neutropenia did not recur. Tian et al. investigated the association between TLRs and the activation and differentiation of T-cells in Takayasu arteritis (TAK). They analyzed the mRNA frequency of 29 target genes in peripheral blood mononuclear cells (PBMCs) from 27 TAK patients and 10 healthy controls. They found increased mRNA levels of TLR2 and TLR4 in TAK patients. Furthermore, this study revealed a novel connection between TLRs and T-cells in the pathogenesis of autoimmune diseases. Another study by Uebelhoer et al. examines how malnutrition and acute illnesses affect immune responses in young children in low- and middle-income countries and which aspects of immunity are relevant in this particularly vulnerable population. Overall, the results show exaggerated innate immune responses to pathogen-associated molecules, especially TLR-4 and TLR-7/8, in acutely ill young children, persisting during recovery. Exaggerated innate immune responses to TLR ligands can contribute to chronic systemic inflammation and dysregulated responses to subsequent infection challenges.

Their ability to stimulate immunity makes TLR attractive targets for expanding numerous immunotherapeutic approaches to combat cancer (15). These immunotherapeutic strategies include the use of TLR ligands/agonists as monotherapy or in combined therapeutic approaches (16). In a review, Chakraborty et al. attempt to provide a comprehensive discussion of significant TLR agonists and their application, as well as the challenges in integrating them into cancer immunotherapy approaches, with a particular emphasis on the use of TLR agonists as functional adjuvants for cancer vaccines. They present the translational potential of an autologous cancer vaccine, as well as the immune-inducing potential of TLR agonists as a potential immunotherapy in various types of cancer.

In another study, Jin et al. show that the unique immune system of the Atlantic cod offers an unprecedented opportunity to explore the evolutionary history of pattern recognition receptor (PRR)-based signaling in the immune defense of vertebrates. The absence of major histocompatibility complex class II antigen presentation and several pathogen recognition receptors in the Atlantic cod has not impaired the immune response.

Overall, this Research Topic effectively illustrates the dual role of Toll-like receptors (TLRs) and their associated signaling pathways in viral infections, as well as in a broad variety of inflammatory processes. It highlights the two facets of TLR activation: one contributing to immune protection and the other to immunopathology.

## Author contributions

OP: Writing – original draft, Writing – review & editing. RK: Writing – original draft, Writing – review & editing.
